# Merkel Cell Carcinoma: A Retrospective Study on 48 Cases and Review of Literature

**DOI:** 10.1155/2012/749030

**Published:** 2012-09-13

**Authors:** Fernando Cirillo, Marco Vismarra, Ines Cafaro, Mario Martinotti

**Affiliations:** ^1^Department of General Surgery, General Surgery Unit, Rare Hormonal Tumors Group, Surgery of Rare Hormonal Tumors, Azienda Ospedaliera Istituti Ospitalieri, Viale Concordia 1, 26100 Cremona, Italy; ^2^Radiotherapy Unit and Nuclear Medicine, Azienda Ospedaliera Istituti Ospitalieri, Viale Concordia, 1, 26100 Cremona, Italy

## Abstract

Merkel cell carcinoma (MCC) is a rare and aggressive neuroendocrine tumor of the skin. Fourty-eight patients with MCC were observed at the Rare Hormonal Tumors Group of Cremona Hospital, 15 of these with unknown primary site. Due to rarity of Merkel cell carcinoma, clinical experience is generally limited. Data from our series confirm the current recommendations. Wide surgical excision must be associated with radiotherapy also in early stages in order to avoid local relapse and the rapid progression of disease. In advanced stages chemotherapy is the standard despite the short duration of responses and poor quality of life. The data of our series, characterized by a high demand for second opinion, offer some insight about the real rarity of the tumor, the difficulty of managing of disease in our country secondary to a wrong cultural approach to the problem, the indiscriminate use of molecules unnecessary and often expensive, the lack of protocols, and the presence of guidelines often ignored. This results in very poor survival associated with a very low quality of life, requiring to find the right direction towards a correct management of disease.

## 1. Introduction

Merkel cell carcinoma (MCC) is a rare and aggressive tumor of the skin described the first time by Toker, in 1972 [1] as a trabecular carcinoma of the skin, suggesting the origin from the cells of the sweat glands, with a later ultrastructural demonstration of neurosecretory granules that places the neoplasm from the Merkel cells [2]. At the beginning of the 1980s, other authors contributed to the discussion on the suitability of the term suggested by Toker, introducing a great variety of alternative names (“primitive small cell carcinoma of the skin,” “malignant Merkel cell carcinoma,” “Murky cell carcinoma,” “Merkeloma”) [3–8]. Finally, histochemical and histogenetic studies coined the term “neuroendocrine carcinoma of the skin” for this neoplasm placing it, therefore, in the large family of APUDomas [9–16], even if the true origin of the tumor from Merkel cell has not been proved definitively. It was also postulated the derivation of Merkel cell from the neural crest, the separation from the cells of Schwann, and the following migration from mesenchyma to epidermis as prolongation of the sensitive nerves of the derma [14]. MCC is currently considered deriving from an epithelial totipotent cell able to differentiate it both in neuroendocrine way, and as cheratinocita [15]. The presence of transitional cells similar both to the cheratinocytis and Merkel cells gives support to this theory.

The definite function of Merkel cell is not clear yet. The nearby contact with sensitive fibers would make to suppose a role of Merkel cell in the process of transition for some nervous stimulus; an influence is also possible on the secretion of neuropeptides as paracrine regulators on the near structures of the epidermis and adnexa [15]. Our observation of a consistent number of cases of MCC has induced us to a review of the literature in order to optimize the diagnostic and therapeutic approach to this tumor, and to emphasize management problems secondary to a cultural limitations that considers in our country MCC like a cancer of little interest with a negative influence in terms of cost and survival.

## 2. Materials and Methods

At the Rare Hormonal Tumors Group, Department of General Surgery of Cremona Hospital, in the last 21 years we have observed 48 patients suffering from MCC in different stages of disease: stage I 28.2%, stage II 8.6%, stage III 26%, and stage IV 37% of cases. Most of the observed patients came from other institutions as a request for second opinion (at least 2/3), and for this reason the analysis of data cannot be homogeneous (Table 1). In order to stage the disease we have preferred to use the previous staging system from American Joint Committee on Cancer (AJCC) 2005 because more simple to use, and because the greatest part of the oldest literature refers to this. 

There were 26 male (54.1%) and 22 female (45.8%) patients with slight male predominance. In our series MCC affects most frequently elderly patients with a mean age of 70.1 for male, and 71.2 for female (male plus female equal to 70.6, range from 52 to 95 years). MCC has been observed as a nodule of the skin in most of the cases. The extremities (40% of cases) were the most common site of incidence: follow unknown primary site (31%), head and neck (19%), buttock (8%), and trunk (1%). Half of cases had lesions with diameter <2 cm, and the other diameter equal to 2 cm or more, with a mean of 2.42 cm, and range from 0.5 to 8 cm. About proliferation cell index (ki67, MIB1 clone), we have separated the series in three different groups (ki67 10–30%, ki67 30–50%, and ki67 > 50%) in which percentage were 13.6% for the group 1, 22.7% for group 2, and 63.6% for group 3 (range from 22% to 90%). The group with the most elevated cell proliferation is prevailing, and this confirms that MCC is a particularly aggressive tumor. In our series we have observed 3 patients with associated rheumatoid arthritis, 3 with hepatitis virus C related, 1 transplanted, 1 with Kaposi sarcoma, and 4 patients with a personal history of tumor (1 non-Hodgkin lymphoma and 3 carcinomas). In 1 case we have observed MCC associated with squamous-cell carcinoma growing together [17]. About unknown primary site, we observed 15 patients (31%): 6 with lesion situated in the groin (40%), 5 in the buttock (33%), and in 1 case lesions were situated in axilla, thigh, vestibule of nose, and parotid gland, respectively.

Role of surgery was confirmed as fundamental for treatment of MCC, above all in early stage. In our series all the patients received surgical approach always: as radical and curative method in early stage, or as debulking for local relapse in advanced stages. Due to the different origin of patients, surgery was associated with other therapies: radiotherapy (12.5%), chemotherapy (10.4%), somatostatin analogues (8.3%), or more treatments together, as radiotherapy plus somatostatin analogues (8.3%), chemotherapy plus somatostatin analogues (6.2%), and radiotherapy plus chemotherapy plus somatostatin analogues (6.2%), radiotherapy plus chemotherapy (2%). In only 4% of cases other treatments were considered, as receptor radionuclide therapy, *α* interferon (IFN), and imiquimod. 

## 3. Results and Discussion

The true incidence of MCC is unknown [18–20]. This tumor most frequently affects elderly patients over the age of sixty (range 7–95) [21], in 78.6% of cases [22], with a preference for women (M : F = 1 : 3) [22, 23]; MCC is most common in Caucasian populations, but occasionally is also present among blacks and Polynesians [15]. The most common site of the tumor is the skin of the head and neck (50%); in 40% of cases extremities are affected, and in 10% trunk and mucosa. Cases have also been reported of multiple sites of the disease [15, 23]. 

The markers normally expressed by this tumour are neuron-specific enolase (NSE) [24], chromogranins [25], and synaptophysin [26]. Vimentin and desmin are usually negative [27, 28]. Cytoplasmatic granules can be rich of vasoactive intestinal polypeptide (VIP), and of met-encephalin.

The neoplasm is typically presented as an isolated, raised or flat lesion, red-purplish in colour, with a shiny surface occasionally associated with nearby telangiectasias. The epidermis may be intact or ulcerated. The tumor can occasionally be pediculate [12, 29]. The size of the neoplasm can vary greatly, up to 15 cm in diameter, with an average of 3 cm at presentation [23]. 

In early stage MCC doesn't present specific characters, so that the differential diagnosis can result difficult: in fact MCC can be confused with the baso or spinocellular carcinoma, the pyogenic granuloma, the cheratoacantoma, the melanoma, the cutaneous linfoma, cutaneous metastasis from anaplastic carcinoma, carcinoid tumors, retinoblastoma, sarcoma of Ewing, and neuroblastoma [15]. A high incidence of the tumor (over 600 cases) was reported in transplanted patients with a mean of 53 years (range 33–78). MCC was observed after 5–286 months from transplant (average 91.5) with characteristics of greater aggressiveness probably secondary to the immunosuppression of the patient [30–32]. The immunosuppressive situation could be the cause of metastatic MCC also in an HIV patient [33]. In our series 3 cases reported of rheumatoid arthritis associated with MCC could be secondary to immunosuppression. Since rheumatoid arthritis is considered an autoimmune disease, it is possible a predisposition to MCC among elderly patients with immune defenses reduced because of the prolonged use of steroid molecules [34].

The staging of MCC considers a whole-body CT spiral scan because of the frequent high-proliferation index and poor differentiation of the tumor, with the aim of identifying metastatic involvement of soft tissues, sometimes associated with lytic bone lesions [35]. Positron Emission Tomography (^18^F-FDG-PET-CT) is an highly useful whole-body-staging method compared to conventional imaging methods, also when used as a single procedure [36, 37]. OctreoScan, using a labelled analogue of somatostatin (^111^In-Pentetreotide), is still considered an highly sensitive method also when compared with other conventional imaging techniques [38]. Laboratory diagnosis considers the plasmatic dosage of chromogranin A and NSE, more specific in posttreatment followup rather than during the stage of the tumor.

Patients affected by MCC can be classified using the last classification AJCC 2010, more online with other skin malignancies, although more complicated to use [39]. Because of this, the literature often refers to the previous staging system from AJCC 2005 [40], more simple to use, but making comparison is difficult with newer studies that consider the last classification. For this reason, we preferred to refer to the classification AJCC 2005 in order to give more homogeneity to our older cases staged by this classification (Table 2).

### 3.1. Surgery

In stage I and II, surgical is the treatment of choice represented by the excision of the primitive lesion [15, 41–44]. In order to avoid local recurrence, an adequate resection margin of at least 2 cm is required [45, 46]. A more wide excision provides a significant reduction in local recurrence rate by increasing the margin from 1 to 3 cm [15, 47, 48]. In our series of 8 cases from other institutions, a wide excision was not considered after histological examination causing a local relapse to distance. The necessity of elective lymph nodal treatment is controversial. Tumor size > 1 cm was found to be a poor prognostic factor [49], and 2 cm can be a significant cut off for poor prognosis [34, 40]. For these reasons, and also in relationship with our experience, we suggest that Sentinel Lymph Node Biopsy (SLNB) should always be considered [50]. SLNB detects MCC spread in one-third of patients understaged, and those who did not receive treatment that involved nodes [51]; this method identifies occult nodal metastases in 29% of patients with localized MCC [52]. About this method, in our series we have observed a higher sensibility using ^18^F-scan rather than ^99^Tc-scan. Finally, in absence of SLNB, adjuvant radiotherapy to the primary and nodal region should be delivered.

### 3.2. Radiotherapy

The greatest part of authors are in favor to consider adjuvant postoperative radiotherapy routinely. This choice is associated with a reduced risk of local recurrence [53, 54]. Radiant treatment (40–60 Gy) should follow surgical excision [55] in order to prevent the progression of disease in stage I and II with development of lymph nodal metastases in 40–73% of the cases and local relapse in 23–60% of the cases [56], with a disease free survival only up to 8 months [45]. In these cases surgical debulking can be associated with more sustained radiant regimes with survival in approximately 60% of cases [15], and a disease free survival from 3 to 30 months (average 8 months) [57]. The largest series from SEER data shows median survival for adjuvant radiotherapy up to 63 months compared with median survival without radiant therapy up to 45 months. Radiotherapy is associated with an increased survival particularly for primary lesions greater than 2 cm [58]. In another series from Canada and Australia, combined surgery and radiotherapy improves both loco-regional control and disease-free survival [49]. On the contrary, adjuvant chemotherapy does not reduce the rate of local relaps nor improve survival [59]. We have observed 10 cases from other institutions with local relapse due to the absence of prior radiant therapy that were in need of surgical debulking.

### 3.3. Radiotherapy Alone

Radiotherapy as primary treatment is essential in cases of locally advanced tumors or invasion of critical structures with difficult resectability. It was reported, a study on 3 cases with complete response after primary radiotherapy, and the absence of local relapses for up to 3 years [60]. In a retrospective study there were no statistical differences and disease-free survival between two different groups (radiotherapy alone versus conventional therapy) [61]. In a series of 50 patients, lymph node radiation alone in metastatic nodes has resulted in a great percentage of local control compared with lymphadenectomy alone on both microscopic and palpable nodes, and no differences for overall survival [62].

### 3.4. Chemotherapy

Advanced disease is characteristic of stage IV. Chemotherapy treatment considers a wide range of molecules used both in monotherapy and in combination, as etoposide, carbo/cisplatin, doxorubicin, dacarbazine, vincristine, cyclophosphamide, and methotrexate. Chemotherapy shows a surprising objective response at beginning of treatment (61%) with a progressive drop during a second (45%), and a third line of therapy (20%) [63] with a very short duration, from 3.5 to 12 months [64, 65]. In the TROG study, synchronous carboplatin/etoposide plus radiation have been achieved high levels of locoregional control and survival [66], in contrast with a retrospective study from the same group [67].

### 3.5. Other Methods

Local infiltration of *α*-2b IFN [68], tumor necrosis factor (TNF) [69], hyperthermia in association with low doses of radiotherapy [70], or radiotherapy with TNF-*α*, IFN *γ*, and melphalan [71], have showed occasional remissions with relatively long, but anecdotal, disease-free survival. Among the immunomodulatory molecules, imiquimod combined with radiotherapy has suggested the possible use effective with a complete response up to 7 months in a case reported of MCC of the head [72]. About somatostatin analogues treatment, there is a few number of reports in literature. In one case of metastatic MCC from our series, the treatment with octreotide showed an immediate objective response with a moderate dose (1 mg/day subcutis), in absence of significant side effects and survival over 10 months from the start of therapy [73]; moreover, in 2 cases observed, OctreoScan was been able to determine a partial regression of local relapse, even before starting treatment with somatostatin analogues. In another case reported of local advanced and recurrent MCC of the head, treatment with lanreotide at the dose of 15 mg intramuscular every two weeks showed a favorable course after 17 months from the start of therapy [74]. In other case of metastatic MCC reported treated with octreotide has been observed a favorable course up to 3 years with a good quality of life [75]. Somatostatin analogues can play a role in the therapy of metastatic MCC, in alternative to chemotherapy, limited to selected cases with mild aggressive disease, and with significant density *in vivo* for somatostatin receptors. In our series, somatostatin analogues represent a wide slice in the treatment of MCC (29% of cases) in different modalities of association. Receptor radionuclide therapy is reported only in one case after relapse from MCC in a elderly patient, with a good response [76]. In our series we have treated only one elderly patient suffering from MCC with ^177^Lu-DOTATATE (1.5 GBq), already submitted to other therapies, and probably in a too advanced stage to consent a response.

## 4. Conclusions

MCC is a highly aggressive cancer of the skin with 30% of mortality. The incidence in USA has increased threefold and became the second common cause of nonmelanoma skin cancer death [77]. The most common features were used to create a simple acronym: AEIOU (asymptomatic/lack of tenderness, expanding rapidly <3 months, immunosuppression, older than 50, and location on an ultraviolet-exposed site). These criteria can allow as a clue in the diagnosis of MCC, with three or more criteria in 89% of cases in a series of 195 patients [78]. In relation to the current knowledge, the correct management for MCC is the aggressive and radical excision of the lesion in its early stage in order to reduce the rate of relapse, and to improve survival. Surgery is the mainstay of treatment for MCC when feasible. Outcome depends mainly on the early and wide excision [79], and on sequential radiotherapy, in order to avoid local relapse and/or progressive disease, as also confirmed from our personal observations. In this way, the role of SLNB is in our opinion fundamental also in stage I, given that size of lesion may not match the malignancy of the tumor. About chemotherapy, its role should be revisited with newer molecules including targeted agents. In this way, coexpression of KIT in a high percentage of MCC suggests an important role in Merkel cell transformation [80], so that the potential use of KIT kinase inhibitor-based therapies, as imatinib, should be also considered in metastatic MCC [81, 82].

The finding that polyomavirus (MCPyV) is frequently present in MCC (69–85% of cases) has been confirmed by several independent groups [83]. The integration of this virus before the tumor development supports a role for polyomavirus in tumorigenesis process [84]. In this way, prophylaxis with vaccination against Merkel cell polyomavirus should be possible in high-risk patients, in the future.

In our opinion, our series highlights a number of interesting aspects. The first concerns the number of patients observed. The great number of patients in our case series can suggest the consideration that the MCC, although considered a low-tumor incidence, it is not so quite rare. The second aspect concerns the cultural approach to the problem. Looking at the cases with advanced disease from other institutions, it is evident that the large number of patients to whom it was not proposed or wide surgical excision, or radiotherapy, or both: thus clearly demonstrates the lack of expertise in the management of MCC, and because of the high aggressiveness of MCC, it is subsequently assumed the highest rate of local relapses or metastastic disease. The third aspect relates to the timing in the management of MCC. We have observed several cases where the choice to remove the primary lesion was made after so many months from the onset of disease, and several cases with long latency between histological diagnosis and subsequent treatment decisions. In one case there was not even the histological examination of the primary lesion and in another even that of relapse. These observations are once again due to the lack of experience for MCC, but also towards a management too superficial in regard to a tumor too underestimated. The fourth point concerns the treatment of metastatic disease. Chemotherapy should be considered at present the standard treatment in advanced disease: but in our series we can observe the frequent use of different molecules (particularly somatostatin analogues) for patients from other institutions, which cannot be considered appropriate to control metastatic disease and even related symptoms. The fifth point relates to the lack of diagnostic and therapeutic protocols, a problem affecting almost the entire management of rare tumors. This question also involves the management of MCC and is highlighted by the large number of second opinion requests. The lack of protocols is partly covered by some guidelines (in Italy by the guidelines from ROL, Rete Oncologica Lombarda) in many cases not known and in many other cases disregarded. The sixth and final point concerns the last classification of MCC, which in our opinion is too complex with the result of a difficult staging, and the consequence of a therapeutic approach to disease not always easy, another reason that makes us still choose the previous staging system from AJCC 2005.

**Figure 1:**
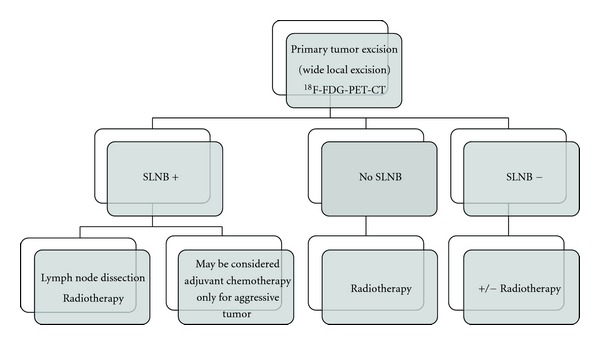
Algorithm for staging and treatment MCC.

**Figure 2:**
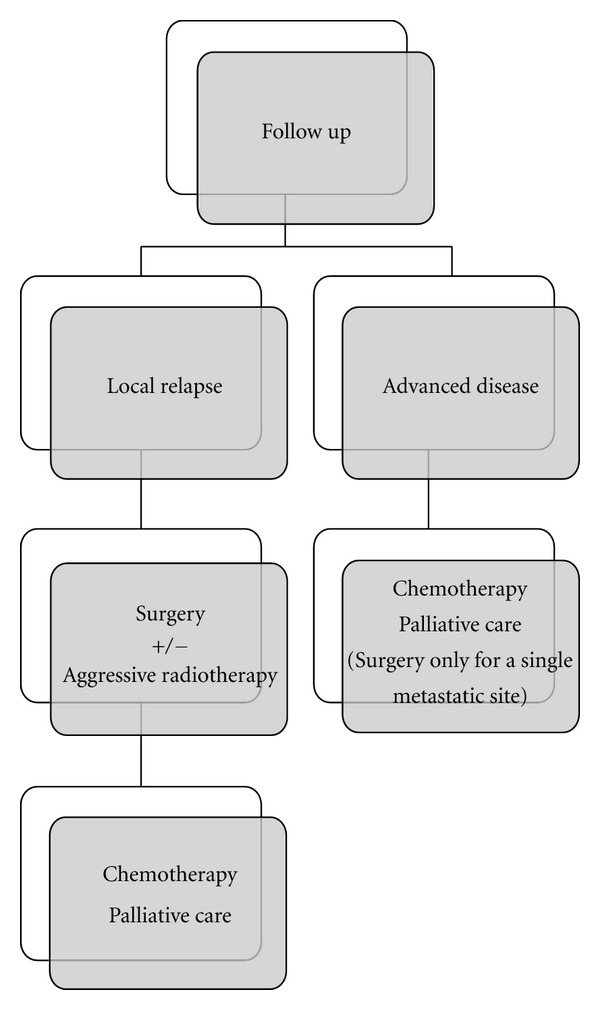
Algorithm for advanced or locally relapsed MCC.

 All these reasons lead clearly to the impossibility having concrete data of survival. In our series the survival rate was calculated considering the distance in time between first diagnosis and our last control of patient. Since the majority of patients we have considered as second opinion in different stages of disease, and the greatest part of these in advanced disease (stage IV) or in presence of local relapse, it is not possible to report the correct data of survival. Furthermore, in a significant part of cases from other institutions we were not able to get further information about the progress of disease. About MCC from unknown primary site (31% in our series), survival appears very low (average 24 months) but conditioned by a very significant late diagnosis up to 18 months, and few treatment options [85]. Finally, we believe that the comprehensive evaluation of the patient integrated with imaging and laboratory parameters can allow to find the right direction for a balanced choice of therapy and not always immediately easy. It will nevertheless require a cultural change in the approach of MCC as in case of other rare tumors (Figures 1 and 2) [50, 86]. 

## Figures and Tables

**Table 1:** Merkel cell carcinoma series (1990–2012).

Sex	Age	Site	Type	Size (cm)	Stage	Ki67% (MIB1)	ChrA staining	NSE staining	ChrA (ng/mL)	NSE (ng/mL)	Therapy	Survival (months)	Other
F	83	EXTR	NOD/ULC	3	III	—	NEG	POS	—	—	SURG + SMS	36	RA
M	52	EXTR	NOD	—	—	—	—	—	—	—	SURG + RT	2	
F	76	BUTTOCK	NOD	3	II	—	NEG	NEG	—	—	SURG	—	
F	75	EXTR	NOD	1	I	30	POS	POS	—	—	SURG + RT	29	RA
M	70	NS	—	3	III	>50	NEG	POS	—	—	SURG + SMS	8	HCV+
F	81	NS	—	1.5	III	—	—	—	—	—	SURG + RT + SMS	26	
F	83	HEAD	NOD	1.2	I	—	NEG	POS	177	5.4	SURG + RT	24	
M	55	BUTTOCK	NOD	3.5	IV	—	—	—	52.2	39.8	SURG + CHT	25	
M	80	TRUNK	NOD	2.2	II	>70	POS	POS	43.2	5.5	SURG + SMS	24	
F	74	HEAD	NOD/CYS	—	I	—	—	—	—	6.3	SURG + RT + SMS + *α* IFN	15	
M	70	EXTR	NOD	0.8	I	—	—	—	—	—	SURG	—	HCV+
F	72	HEAD	NOD	1	I	80	—	—	—	—	SURG	—	Ca breast, Ca lung
F	63	NS	—	5	III	—	—	POS	51	31.3	SURG	25	HCV+ Ca uterus
M	74	EXTR	NOD	1.5	I	—	POS	—	136	5.4	SURG	3	
M	70	NS	—	—	IV	—	—	—	—	—	SURG + CHT	12	
M	85	EXTR	NOD	8	III	25	POS	—	870	43	SURG + SMS	37	Kaposi sarcoma
F	61	EXTR	NOD	4.3	II	80	POS	POS	—	—	SURG	—	
M	76	HEAD	NOD	1.5	III	—	POS	POS	185	8.3	SURG	—	Ca rectus
F	84	EXTR	NOD	2.0	IV	—	POS	POS	—	—	SURG + CHT	48	
F	79	EXTR	NOD	1.5	I	—	—	—	—	—	SURG + RT	—	
M	76	EXTR	NOD	—	IV	—	—	—	—	—	SURG + CHT	12	
F	72	NS	—	4.5	IV	80	POS	—	70	81.2	SURG + CHT + SMS	58	
M	55	EXTR	NOD	1	III	—	NEG	—	—	—	SURG	—	
F	80	EXTR	NOD/ULC	1.7	I	80	POS	POS	—	—	SURG	—	
M	67	HEAD	NOD	0.5	I	—	—	—	—	—	SURG	—	
M	70	NS	—	—	IV	70	—	—	46	13	SURG + CHT	16	
M	70	EXTR	NOD	2	IV	40	—	—	99.3	17.8	SURG + RT + CHT + SMS	27	
F	61	NS	—	6	IV	50	—	—	50	21.3	SURG + CHT	—	
M	63	BUTTOCK	NOD	5	IV	50	POS	—	—	—	SURG	15	LNH
M	95	HEAD	NOD	1.2	I	70	POS	—	—	—	SURG	—	
M	80	HEAD	NOD	0.5	IV	—	—	—	46	8.9	SURG + RT + CHT + SMS + RMT	17	
M	74	NS	—	—	IV	—	—	NEG	—	—	SURG + RT + CHT + SMS	23	
F	69	EXTR	NOD	—	IV	—	—	—	—	—	SURG	—	
M	64	NS	—	3	II	60	—	POS	156	8.0	SURG	—	
F	60	HEAD	NOD	0.7	I	90	POS	POS	—	—	SURG	—	
M	89	BUTTOCK	NOD	—	IV	80	POS	—	760	86.2	SURG + RT + CHT	13	
F	65	EXTR	NOD	—	—	—	—	—	—	—	SURG	—	
M	59	EXTR	NOD	1	III	—	POS	POS	—	—	SURG	6	
M	64	HEAD	NOD	1.1	III	60	POS	—	—	—	SURG	—	RA
F	59	EXTR	NOD	0.6	I	22	—	—	—	—	SURG	17	
M	75	NS	—	4	IV	—	—	—	—	—	SURG	6	HCV+
F	59	NS	—	1	I	40	POS	—	—	—	SURG	14	
F	78	EXTR	NOD	2.5	III	—	—	—	—	—	SURG	—	
F	60	NS	—	—	IV	—	—	—	—	—	SURG	—	Paraneoplastic polineuritis
M	69	EXTR	NOD	2	III	35	POS	POS	116	10	SURG + RT	5	Transpl
F	74	NS	—	6	IV	—	POS	—	700	102	SURG + CHT+ SMS	52	
M	58	NS	—	2.5	III	80	POS	POS	46.5	5.3	SURG + RT	—	
M	63	NS	—	1.2	IV	80	POS	—	1500	17.20	SURG + CHT + SMS	22	

AR: rheumatoid arthritis, ChrA: chromogranin A, 19–98 ng/mL, CHT: chemotherapy, EXTR: extremities, F: female, *α* IFN: alpha interferon, M: male, NS: no skin (unknown primary site), NSE: neuron-specific enolase, <12 ng/mL, RM: receptor radionuclide therapy, RT: radiotherapy, SMS: somatostatin analogues, SURG: Surgery, TRANS: transplanted.

**Table 2:** Merkel cell carcinoma staging system, 2005 [40].

Stage	TNM	OS 2 y	OS 5 y
Stage I	Primary < 2 cm (T1)	67%	81%
Stage II	Primary 2 cm or more (T2)	59%	67%
Stage III	Nodal disease (N1)	49%	52%
Stage IV	Systemic metastases (M1)	23%	11%
